# Hypertrophic scar mimicking peristomal pyoderma gangrenosum^☆^

**DOI:** 10.1016/j.abd.2022.07.012

**Published:** 2023-12-14

**Authors:** Takashi Ito, Toshiyuki Yamamoto

**Affiliations:** Department of Dermatology, Fukushima Medical University, Hikarigaoka, Fukushima, Japan

Dear Editor,

Peristomal Pyoderma Gangrenosum (PPG) is a subtype of pyoderma gangrenosum, arising around the stoma after surgical placement of an ileostomy or colostomy in patients with inflammatory bowel diseases.^1^ Because there are a number of skin disorders involving the peristomal or parastomal areas, PPG may be overdiagnosed.^2^ We herein describe an unusual case presenting with hyperkeratotic lesions around the stoma in a patient after colorectal cancer surgery.

A 78-year-old male after colorectal cancer surgery was referred to us, complaining of hypertrophic lesions surrounding the stoma. He received a colostomy 6-months previously, and peristomal skin lesions gradually worsened in these 2-months. He suffered from exudate from the lesions and pain associated with skin infections. Physical examination showed relatively well-circumscribed vegetating and keratotic lesions around the lower left abdominal stoma (Fig. 1). No abnormalities were found in the blood test.

**Figure 1:**
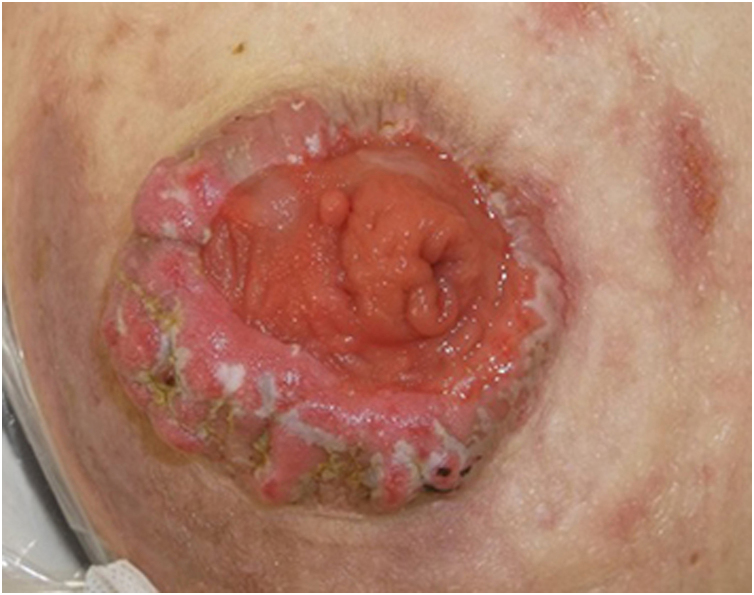
Hyperkeratotic lesions presenting with well-circumscribed vegetating and keratotic appearances surrounding the stoma.

Histological features showed irregular hypertrophy of the epidermis, with dilated blood vessels in the papillary dermis and edematous upper dermis (Fig. 2A). Neutrophil infiltration and histological malignancy were not observed. Immunohistochemistry showed dense staining for vimentin in the mesenchymal cells in the dermis (Fig. 2B). CD31 staining showed a number of vessels, and CD31-positive vascular endothelial cells were observed throughout the dermis (Fig. 2C). α-Smooth Muscle Actin (SMA)-positive myofibroblasts were proliferated (Fig. 3A), which were partially positive for p16 (Fig. 3B). A diagnosis of hypertrophic scar was made, and the patient received reoperation of the stoma including the surrounding skin lesions.

**Figure 2:**
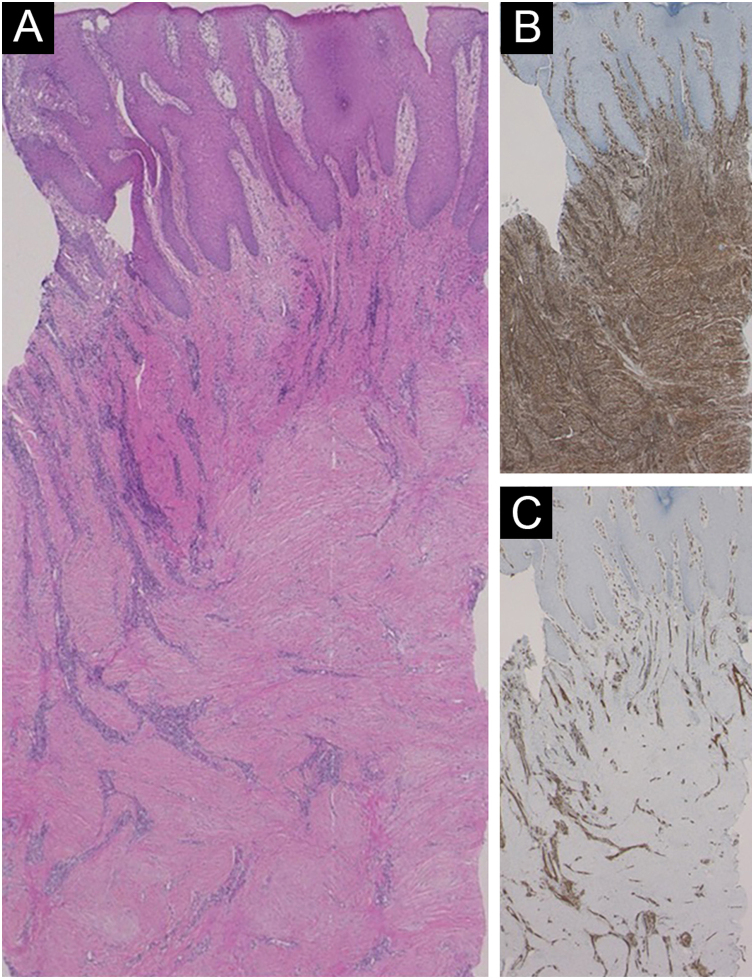
(A) Histopathological features showing fibrosis of the thickened dermis. (B) Immunohistochemical examination using anti-vimentin antibody showed proliferation of mesenchymal cells. (C) The vessels in the dermis were increased in number, which were positive for CD31.

**Figure 3:**
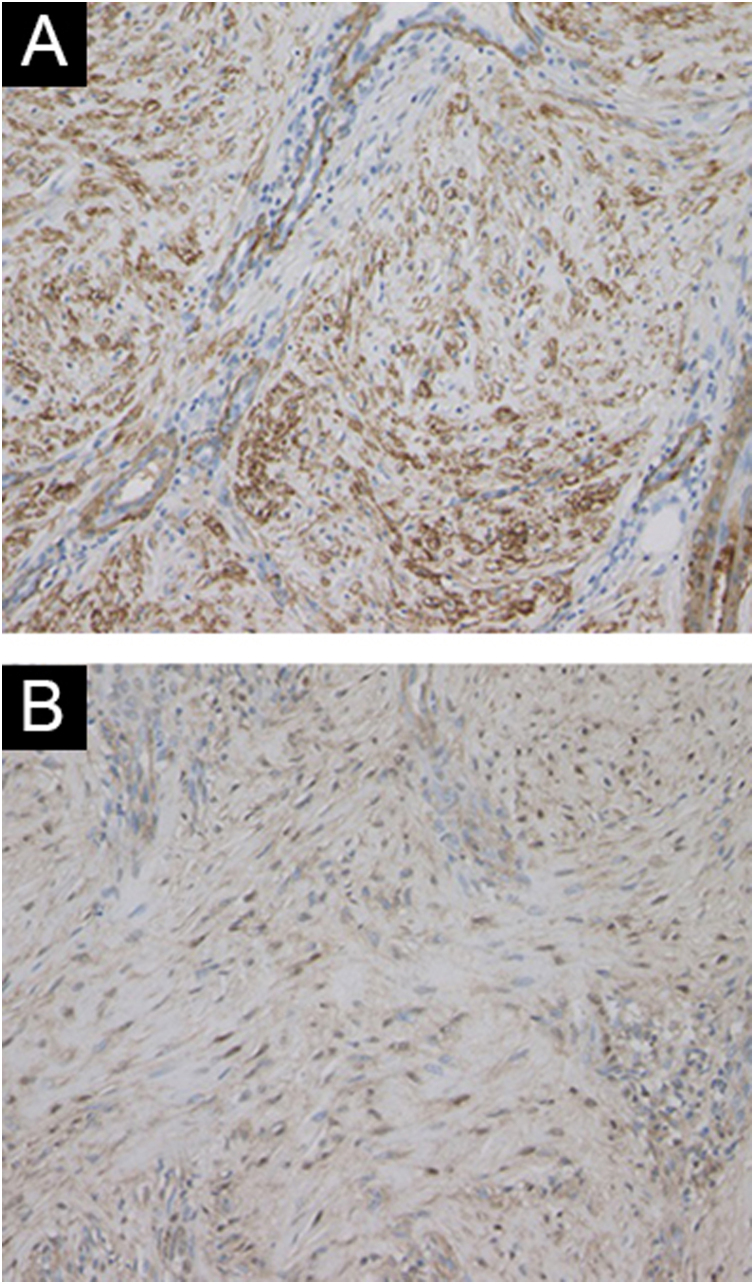
Myofibroblasts were increased in number, which were positively stained with α-SMA (A), and partially positive for p16 (B).

Peristomal pyoderma gangrenosum is a subtype of pyoderma gangrenosum, arising around the stoma in patients with inflammatory bowel diseases, and is observed in around 1% of patients with stoma.^1^ By contrast, it is also suggested that PPG is overdiagnosed from its clinical features.^2^ Currently, there are no standard diagnostic criteria, and there are several conditions that should be differentiated from PPG. Those conditions include irritant and contact dermatitis, infection, overgranulation, pseudo verrucous lesion, and squamous cell carcinoma.^3^ In addition, other reports showed two cases of peristomal ulcerative conditions, which were eventually reclassified to be caused by irritant dermatitis;^4^ however, the hypertrophic scar was not included. The present case did not present with surrounding ulcers but hyperkeratotic lesions around the stoma. Histopathological examination did not reveal neutrophil infiltration in the dermis, but increased, thickened, and whorled collagen bundles, and a number of CD31-positive vessels throughout the dermis. Recent studies demonstrated strong expression of vimentin, α-SMA, and p16 in the hypertrophic/keloid scars, suggesting the proliferation of cellular senescence phase myofibroblasts.^5^ We should keep in mind that a number of inflammatory conditions assume clinical appearance mimicking PPG, and careful differentiation from other disorders is necessary for accurate diagnosis of PPG.

## Financial support

None declared.

## Authors’ contributions

Takashi Ito: Conducted the dermatological examination and treatment of the patient, and wrote a draft of the manuscript.

Toshiyuki Yamamoto: Substantial contribution for interpretation, revision, and final approval.

## Conflicts of interest

None declared.
